# Predicting prognosis prior to the combination of atezolizumab and bevacizumab on unresectable HCC: Analysis and comparison of tumor heterogeneity at CT and Gd-EOB-DTPA hepatobiliary MR imaging

**DOI:** 10.1097/MD.0000000000040769

**Published:** 2024-12-06

**Authors:** Heejin Kwon, Eunju Kang, Sanghyun Kim, Yanghyun Baeck, Ilcheol Bark, Jinhan Cho

**Affiliations:** a Department of Radiology, Dong-A University College of Medicine, Busan, Republic of Korea; b Department of Internal Medicine, Dong-A University College of Medicine, Busan, Republic of Korea.

**Keywords:** chemotherapy, CT, HCC, MR

## Abstract

Since 2007, the combination of atezolizumab and bevacizumab, comprising an immune checkpoint inhibitor and a molecularly targeted agent, has become the first-line treatment for advanced hepatocellular carcinoma (HCC). Predicting prognosis prior to systemic chemotherapy remains a critical concern. This study included 84 advanced HCC patients who underwent enhanced computed tomography (CT) and Gd-EOB-DTPA magnetic resonance imaging (MRI) before the systemic therapy were included. In CT, the 2 radiologists measured mean CT Hounsfield unit (CTHU) value by drawing region of interest at the largest diameter of the tumor on arterial phage. The HU values were categorized into 5 groups: ≤ 0, 0 < HU ≤ 50, 50 < HU ≤ 100, 100 < HU ≤ 150, and HU > 150. The percentage of the entire tumor in each category was calculated. On MRI, hepatobiliary phase imaging features and relative enhancement ratio (RER) were also evaluated by 2 radiologists. Prognostic factors associated with progression-free survival were identified using statistical analysis. RER on HBP MRI correlated with prognosis in systemic chemotherapy. Conversely, other image features on HBP MRI and CT histogram provided consistent treatment effect.

## 1. Introduction

Hepatocellular carcinoma (HCC) is the most common type of primary hepatic malignancy in adult patients and the second leading cause of cancer-related mortality.^[1]^ Surgical treatment is indicated only in the very early stages, while HCC is often diagnosed in the more advanced stage. The overall outcomes of patients with advanced HCC remain unsatisfactory, and there are limited therapeutic options of the majority with advanced stage.^[2]^ Currently, molecular targeted agents and immune checkpoint inhibitors (ICI) have emerged as the new standard of care for HCC patients. Phase III trials in patients with unresectable HCC have demonstrated the significant efficacy of atezolizumab in combination with bevacizumab over sorafenib, the first approved targeted agent for advanced HCC, in terms of overall survival and progression-free survival (PFS).^[3]^ Consequently, this combination therapy has ascended to the forefront as the first-line treatment for advanced HCC.^[4,5]^

With a variety of systemic treatment options variable, accurate assessment of therapeutic response to predict efficacy before the atezolizumab plus bevacizumab combination therapy is important to treatment planning. This approach can guide the decision to pursue early re-treatment to attain stable disease in responders; conversely, it may expedite the transition to alternative treatment modalities in nonresponders.

Despite, the promising results of atezolizumab plus bevacizumab therapy for advanced HCC, response often is mixed. Therefore, identifying factors that can predict treatment response before initiating the combination therapy is crucial.

Recently, Aoki et al, suggested that advanced HCC with isointensity or hyperintensity on hepatobiliary phase (HBP) had a low response rate to ICI.^[6]^ In other study, Kurebayashi et al reported that majority of HCCs presenting with the macrotrabecular-massive HCC and vessel encapsulating tumor cluster type were immune cold, resulting in a poor prognosis.^[7]^ Image findings indicative of these tumor types have been reported to include intratumoral hemorrhage and necrosis, as well as sizes larger than 5cm on contrast-enhanced computed tomography (CT) scans.

In this study, we conducted an analysis of tumor heterogeneity through histogram analysis using enhanced CT to predict the response of HCC to atezolizumab plus bevacizumab therapy. Additionally, we evaluated the hepatobiliary phase (HBP) in Gd-EOB-DTPA magnetic resonance imaging (MRI) in the same patients and compared it with CT to assess the feasibility of predicting the treatment effect of HCC.

## 2. Patients and methods

This study is a retrospective analysis of data collected as part of a prospective study, which was approved with our institutional review board (DAUHIRB-24-150) and with written informed consent of all patients included in this study.

### 2.1. Study population

**Figure 1.**
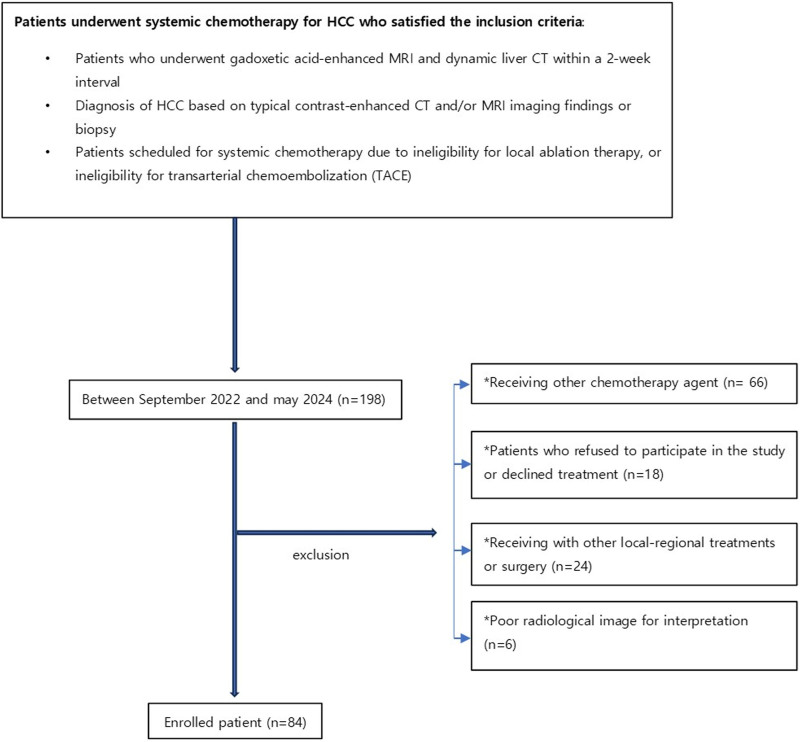
Flowchart of patient selection.

Between September 2022 and May 2024, 84 patients with advanced HCCs were consecutively enrolled in this study. Inclusion criteria were as follows: patients with underlying liver cirrhosis and a confirmed diagnosis of advanced HCC; diagnosis of HCC based on typical contrast-enhanced CT and/or MRI imaging findings, characterized by arterial enhancement followed by washout on portal venous or delayed phase images, or confirmed by biopsy; patients who underwent gadoxetic acid-enhanced MRI and dynamic liver CT within a 2-week interval; patients scheduled for systemic chemotherapy due to unresectability, ineligibility for local ablation therapy, or ineligibility for transarterial chemoembolization (TACE). Exclusion criteria were as follows: patients receiving with other treatments or agents; patients who refused to participate in the study or declined treatment altogether. Figure 1 provides a summarized flow chart of the study.

### 2.2. Treatment and evaluation criteria for response

Intravenous administration of 1200 mg atezolizumab combined with 15 mg/kg of bevacizumab was conducted every 3 weeks. Treatment discontinuation occurred upon the observation of unacceptable adverse events or clinical tumor progression. Response to treatment was assessed using contrast-enhanced CT or MRI in accordance with the response evaluation criteria in solid tumors^[8,9]^ every 8 to 12 weeks. The optimal response observed was considered the therapeutic effect.

### 2.3. Image analysis

#### 2.3.1. Observation registration

A board-certified abdominal radiologist with 10 years of liver imaging experience identified consecutive cases that met the inclusion and exclusion criteria using a picture archiving and communication system. For each selected patient, the size and location of the observations were reported, and the 2 largest lesions were chosen for further image analysis. Subsequently, 2 board-certified radiologists with over 15 and 8 years of experience in liver imaging, respectively, reviewed the selected observations according to the imaging analysis protocol described below.

#### 2.3.2. CT image analysis

Arterial phase CT images were transferred from the online medical image database in DICOM format, and the transfer was confirmed. Registration images were then created using AquariusNET Viewer software (version 4.4.13, TeraRecon). The images were reformatted into axial views, and the largest axial image containing the target HCC was selected. Using 2D image analysis histogram software, regions of interest were manually delineated around the 2 largest lesions to assess heterogeneity. Mean CT attenuation in Hounsfield units (CTHU) was analyzed, and values for mean-HU, min-HU, max-HU, and standard deviation (SD)-HU were simultaneously calculated for each ROI. The HU histogram analysis (HUHA) was categorized into 5 ranges: A ≤ 0, 0 ≤ B < 50, 50 ≤ C < 100, 100 ≤ D < 150, and 150 ≤ E, with each value expressed as a percentage of the total ROI area.

#### 2.3.3. MR image analysis

Liver MRI was performed using a 3.0-Tesla system (Discovery MR750, GE Healthcare, Waukesha, WI, and MAGNETOM Vida, Siemens Healthcare). Dynamic contrast-enhanced imaging was conducted using a 3D-spoiled gradient-echo sequence, with a 15-second breath-hold before and after injecting 0.1 mL/kg of gadoxetic acid (Primovist; Bayer Healthcare) at 1 mL/s, followed by a 20 mL saline flush at the same rate. Post-contrast dynamic T1-weighted hepatobiliary phase (HBP) images were acquired 20 minutes after injection, with a slice thickness of 5 mm and a spacing of 2.5 mm. EOB-MRI images of HCC lesions were independently assessed by 2 radiologists, categorizing each lesion as either homogeneous or heterogeneous based on visual assessment. Any discrepancies in imaging features were resolved through discussion, resulting in a consensus categorization. Additionally, signal intensity (SI) for both tumor lesions and non-tumor liver tissue was measured by defining an ROI, following previously reported methods.^[10]^ The relative enhancement ratio (RER) was calculated using the formula: (nodule SI/parenchyma SI on hepatobiliary phase images)/(nodule SI/parenchyma SI on pre-contrast images). The tumor contour and the presence of portal vein tumor thrombus were also evaluated.

### 2.4. Statistical analysis

We utilized commercially available statistical software, specifically SPSS version 23.0 (IBM Corp., Armonk, NY, USA) and MedCalc version 16.4 (MedCalc Software bvba, Mariakerke, Belgium), for data analysis. A 2-sided *P*-value of < .05 was considered statistically significant.

Inter-reader agreement for CT and MRI images was assessed using intraclass correlation coefficients (ICC), with the following thresholds: < 0.20 indicating poor agreement, 0.20 to 0.39 fair agreement, 0.40 to 0.59 moderate agreement, 0.60 to 0.79 substantial agreement, and > 0.80 excellent agreement. Quantitative parameters were compared using independent-samples *t*-tests or the Mann–Whitney *U* test, depending on data distribution. Chi-square tests were employed for categorical data analysis. The correlation between categorical HU values and patient PFS was assessed using a correlation coefficient. For the RER on hepatobiliary phase Gd-EOB-DTPA MR images, the Kaplan–Meier method and log-rank test were used to estimate PFS following drug administration.

## 3. Result

### 3.1. Patient characteristics and response to atezolizumab plus bevacizumab therapy

The baseline characteristics of the 84 patients enrolled in this study are summarized in Table 1. The observation periods after treatment with atezolizumab plus bevacizumab therapy were from 1month to 19 months (median: 10 months).

**Table 1:** Characteristics of the study population.

Variables	Male (n = 63)	Female (n = 21)	Total (n = 84)
Age (yr)			
Mean ± SD	62.4 ± 7.2	65.1 ± 9.8	63.8 ± 8.9
Range	39 to 81	52 to 89	39 to 89
Etiology of cirrhosis, n (%)			
HBV	23 (36.5)	7 (33.3)	30 (35.7)
HCV	18 (28.6)	2 (9.5)	20 (23.8)
Alcohol	10 (15.9)	3 (14.3)	14 (16.7)
HBV + HCV	–	3 (14.3)	1 (1.2)
Alcohol + HBV	4 (6.3)	5 (23.8)	5 (6.0)
Alcohol + HCV	6 (9.5)	–	6 (7.1)
Alcohol + HBV + HCV	1 (3.2)	–	1 (1.2)
Autoimmune hepatitis	–	1 (4.8)	1 (1.2)
Cryptogenic	1 (3.2)	2 (9.5)	3 (3.6)
MELD score			
Mean ± SD	9.8 ± 3.1	9.2 ± 2.8	9.7 ± 3.5
Range	7 to 16	7 to 17	7 to 17
Treatment period			
Month			10 (1–19)

Abbreviations: HBV = hepatitis B virus, HCV = hepatitis C virus, MELD = model for end-stage liver disease, SD = standard deviation.

### 3.2. HCC image analysis by arterial histogram of CT

Regarding histogram analysis according to categories, the weighted κ value for agreement between the 2 observers were 0.849 to 0.999 (excellent agreement). Regarding the mean-HU, min-HU, max-HU, SD HU values for agreement between the 2 observers were κ: 0.646 (substantial agreement) ~ κ: 0.998 (excellent agreement).

### 3.3. Relationship between treatment response and assessment of arterial phase histogram on CT image

**Figure 2.**
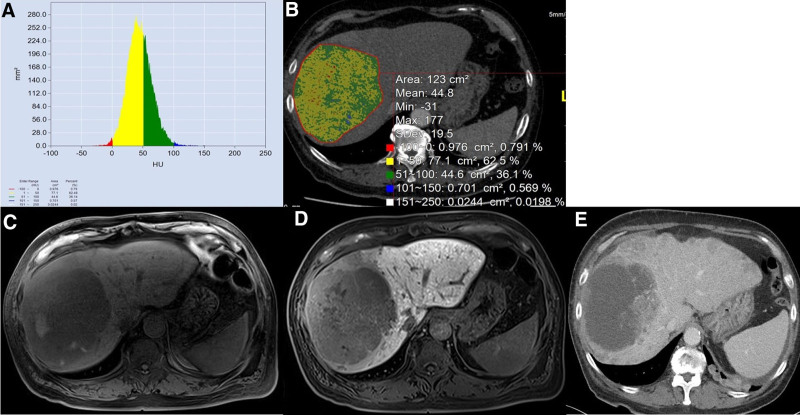
69-yr-old male patients with hepatocellular carcinoma not responding to atezolizumab plus bevacizumab. Computed tomography histogram analysis before atezolizumab plus bevacizumab (A and B) depicts relatively homogeneous vascularized HCC with mean-Hounsfield unit of 44.8, SD 19.5 Hounsfield unit measured in a region of interest covering the largest lesion. On EOB-DTPA pre-enhanced T1 weighted image (C) and hepatobiliary phase (D) the relative enhancement ratio ≥ 0.9 was defined. After 3 months of atezolizumab plus bevacizumab therapy, the new intrahepatic lesions are appeared, consistent with progression disease according to mRECIST (E). HCC = hepatocellular carcinoma.

The mean arterial phase (AP) values and SDs of target lesions did not show a significant difference in patients’ PFS, disease control rate (DCR), or objective response rate (ORR) (ρ > 0.05). Pearson correlation analysis revealed no significant association between PFS and the HU ranges: 0 < HU ≤ 50, 50 < HU ≤ 100, 100 < HU ≤ 150, and HU > 150. However, a weak correlation was identified for HU ≤ 0 with PFS (ρ = 0.040, 0.047). Additionally, the percentage of intratumoral HU ≤ 50 showed no significant correlation with PFS, as indicated by reader 1 (*r* = 0.255; ρ = 0.107) and reader 2 (*r* = −0.232; ρ = 0.145) (Table 2 and Fig. 2).

**Table 2:** Progression-free survival stratified by assessment of CT histogram.

CT histogram (reader 1)				CT histogram (reader 2)			
Variables		Progression-free survival (mo)		Variables		Progression-free survival (mo)	
		correlation coefficient	ρ			correlation coefficient	ρ
	Total area	0.104	0.519		Total area	0.114	0.476
	Mean-HU	–0.342	0.029		Mean-HU	–0.341	0.029
	Min	0.010	0.951		Min	–0.059	0.715
	Max	–0.293	0.063		Max	–0.217	0.172
	Sdecv	–0.250	0.114		Sdecv	–0.104	0.516
≤0 (HU)	Area (cm^2^)	0.182	0.255	≤0 (HU)	Area (cm^2^)	0.404	0.009
	Percent (%)	0.323	0.040		Percent (%)	0.312	0.047
1 to 50	Area (cm^2^)	0.038	0.813	1 to 50	Area (cm^2^)	0.044	0.784
	Percent (%)	0.251	0.113		Percent (%)	0.227	0.154
51 to 100	Area (cm^2^)	0.169	0.292	51 to 100	Area (cm^2^)	0.184	0.251
	Percent (%)	0.133	0.408		Percent (%)	0.164	0.305
101 to 150	Area (cm^2^)	–0.020	0.899	101 to 150	Area (cm^2^)	–0.017	0.914
	Percent (%)	–0.294	0.062		Percent (%)	–0.303	0.054
151<	Area (cm^2^)	–0.144	0.368	151<	Area (cm^2^)	–0.133	0.407
	Percent (%)	–0.215	0.178		Percent (%)	–0.208	0.192

### 3.4. HCC image analysis by EOB-DTPA-MRI hepatobiliary phase

Regarding the visual assessment of hepatocellular carcinoma (HCC), the weighted kappa (κ) values for inter-observer agreement on homogenous vs heterogeneous, nodularity vs infiltrative pattern, and the presence of portal vein thrombosis ranged from 0.8233 to 1.000, indicating excellent agreement. For the ratio of enhancement ratio (RER) assessment, the intraclass correlation coefficient (ICC) for inter-observer agreement was 0.790, signifying substantial agreement. Visually, the heterogeneous type constituted 29% of cases, the infiltrative type 36.6%, and portal vein thrombosis was present in 56% of cases. Additionally, in the RER assessment, 63.3% of the cases had an RER of <0.9.

### 3.5. Relationship between treatment response and assessment of EOB-DTPA-MRI hepatobiliary phase

**Figure 3.**
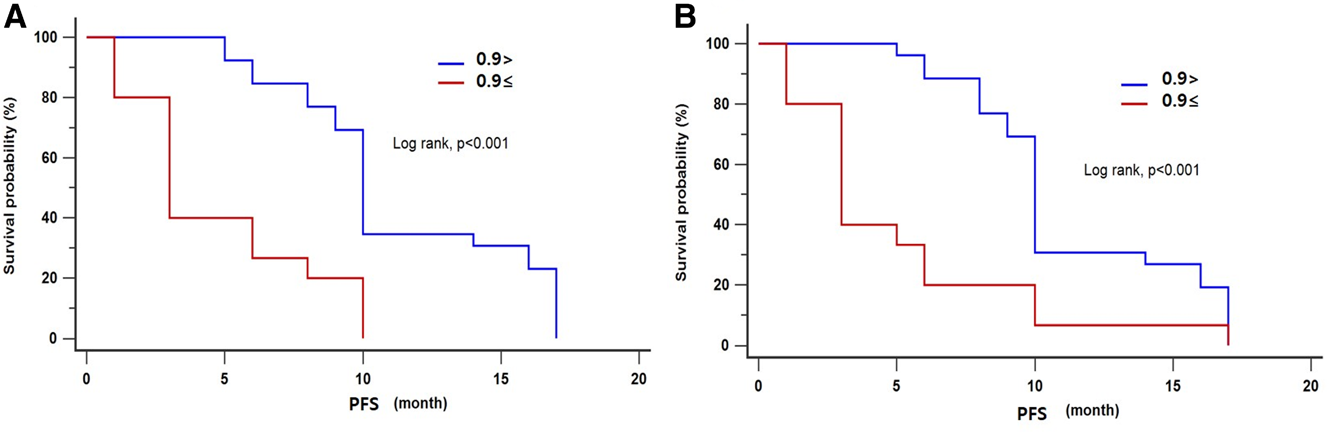
Progression-free survival stratified by relative enhancement ratio in EOB-MRI. Kaplan–Meier curve of progression-free survival in reader 1 (a) and reader (2). Progression-free survival was significantly better in the hypointensity type (*R* < 0.9) than in the hyperintensity type (ρ < 0.001, log-rank test) at both readers.

**Figure 4.**
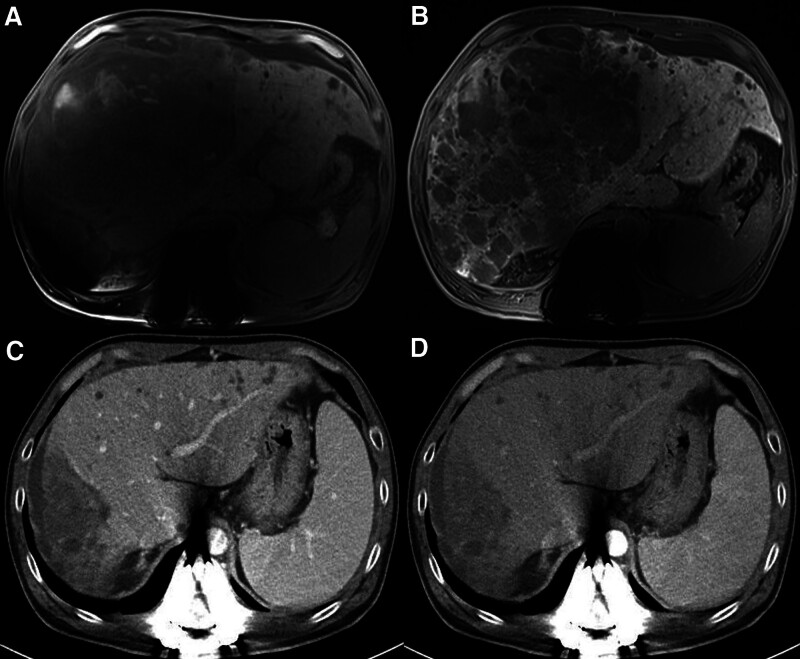
48-yr-old male patients with hepatocellular carcinoma responding to atezolizumab plus bevacizumab. On EOB-DTPA pre-enhanced T1 weighted image (A) and hepatobiliary phase (B), the relative enhancement ratio < 0.9 was defined. After 17 mo of atezolizumab plus bevacizumab therapy, the lesion became shrinkage and decreased in size, consistent with partial response according to RECIST (C and D).

Visual analysis of imaging findings to determine whether a tumor is heterogeneous, has indistinct margins, or is accompanied by portal vein tumor thrombus did not demonstrate a statistically significant difference in patients’ PFS, DCR, and ORR (ρ > 0.05). However, in the ratio of enhancement ratio (RER) assessment, there was a significant difference in PFS between the groups with RER < 0.9 and RER ≥ 0.9 (ρ = 0.01) (Table 3, Figs. 3 and 4), as well as in DCR and ORR (ρ = 0.015) (Table 4).

**Table 3:** Progression-free survival stratified by assessment of EOB-MRI hepatobiliary phase.

		Reader 1				Reader 2			
		Progression-free survival (mo)		*p*		Progression-free survival (mo)		*p*	
		Mean ± SD	Median (IQR)	*t*-Test	Mann–Whitney *U* test	Mean ± SD	Median (IQR)	*t*-Test	Mann–Whitney *U* test
RER	<0.9	11.31 ± 4.15	10.00 (9.00–16.00)	**0.01**	**0.01**	11.15 ± 3.84	10.00 (9.00–16.00)	**0.01**	**0.01**
	<0.9	4.73 ± 3.35	3.00 (3.00–8.00)			5.00 ± 4.38	3.00 (3.00–6.00)		
Type	Homogeneous	9.66 ± 5.43	10.00 (5.00–16.00)	0.135	0.206	9.43 ± 5.47	10.00 (5.00–16.00)	0.266	0.386
	Heterogeneous	7.08 ± 3.26	8.00 (4.50–10.00)			7.45 ± 3.14	8.00 (6.00–10.00)		
Pattern	Nodular	9.04 ± 5.17	9.50 (5.00–10.00)	0.822	0.934	9.04 ± 5.17	9.50 (5.00–10.00)	0.822	0.934
	Infiltrative	8.67 ± 4.84	10.00 (5.00–10.00)			8.67 ± 4.84	10.00 (5.00–10.00)		
PV thrombosis	X	7.89 ± 4.54	8.00 (6.00–10.00)	0.255	0.323	7.89 ± 4.54	8.00 (6.00–10.00)	0.255	0.323
	O	9.70 ± 5.29	10.00 (5.00–16.00)			9.70 ± 5.29	10.00 (5.00–16.00)		

Bold indicates statistically significant values having *P* < .05.

**Table 4:** Response rate stratified by assessment of EOB-MRI hepatobiliary phase.

		Reader 1		Reader 2	
	Chi-square p	Disease control rate (*P*-value)	Objective control rate (*P*-value)	Disease control rate (*P*-value)	Objective control rate (*P*-value)
RER	<0.9	**.01**	**.015**	**.01**	**.015**
	≥0.9				
Type	Homogeneous	.128	.084	.247	.152
	Heterogeneous				
Pattern	Nodular	.300	.218	.300	.218
	Infiltrative				
PV thrombosis	X	.613	.529	.613	.529
	O				

Bold indicates statistically significant values having *P* < .05.

## 4. Discussion

It has been shown that ICI and molecular target agents are effective in many cancers including HCC. However, only a few studies so far have evaluated the biomarkers can be used to predict their therapeutic effects of atezolizumab plus bevacizumab therapy.

Tumor heterogeneity has traditionally been recognized as a critical prognostic factor in cancer treatment, correlating with higher tumor grades, poorer treatment responses, increased likelihood of metastasis, and shorter PFS.^[11,12]^ Tumor heterogeneity arises from variations in vascularization, cell density, necrosis, and fibrosis within the tumor.^[13]^ Despite the well-established significance of tumor heterogeneity, no studies to date have directly examined whether CT histogram analysis of tumor heterogeneity can predict treatment response in patients receiving atezolizumab plus bevacizumab therapy.

The hepatobiliary phase of gadoxetic acid-enhanced MRI (EOB-MRI) has been reported to be useful as an imaging biomarker for detecting β-catenin mutations.

Fujita^[14]^ and Kubo^[15]^ studies reported that the heterogeneous type assessed by the EOB-DTPA MRI hepatobiliary phase was approximately 40%, and the hyperintensity type was approximately 20% in large size lesions in patients with unresectable HCC. In Sasaki study,^[16]^ most cases of the homogeneous type identified by visual assessment were hyperintensive (RER ≥ 0.9), but among the heterogeneous type, the hyperintensity type (RER ≥ 0.9) was 42.9%.

Another main finding is that heterogeneous type and the hyperintensity type (RER ≥ 0.9) type had significantly shorter PFS than homogeneous and hypointensity type in the atezolizumab plus bevacizumab therapy.

In the present study, we compared pretreatment CT histograms and EOB-DTPA MR images to evaluate the potential of imaging markers in predicting the response to atezolizumab plus bevacizumab therapy. Among the various imaging findings analyzed, only hyperintensity (RER ≥ 0.9) in the hepatobiliary phase of EOB-DTPA MRI was uniquely associated with poor treatment outcomes in patients undergoing this therapy. One possible explanation for this predictive ability is the association of the hyperintense type with a subset of HCC patients harboring β-catenin mutations. However, it is important to note that the prediction rate of β-catenin mutation using EOB-DTPA MRI is approximately 80%,^[17]^ indicating that additional factors may contribute.

The hyperintense type also tends to be associated with larger tumor size, which may reflect the degree of differentiation within HCC and the heterogeneity of its molecular biological characteristics. It is noteworthy, however, that disease control was achieved in some cases with hyperintense type tumors following atezolizumab plus bevacizumab therapy. This could be attributed to bevacizumab, an antibody targeting VEGF-A,^[18]^ which promotes T-cell infiltration and transforms suppressive immune microenvironments into responsive ones. These findings suggest that atezolizumab plus bevacizumab therapy could still be effective even in the presence of WNT/β-catenin mutations.

This study has several limitations. First, the study was presented at a single institution, which may have introduced bias due to the specific patient population and the experience level of the radiologists involved. Second, we included only patients who had undergone both CT and MRI within a 2-week interval, resulting in the exclusion of many cases. Third, many of our observations were confirmed through follow-up imaging rather than pathological diagnosis, which is an inherent limitation given that imaging is generally preferred at this stage of disease management. Nonetheless, we applied strict reference standards and included only cases with clear clinical and imaging features.

Despite these limitations, this study is the first to compare CT and Gd-EOB-DTPA MRI in predicting the therapeutic effects of atezolizumab plus bevacizumab therapy in patients with unresectable HCC. In conclusion, hyperintensity in the hepatobiliary phase of EOB-DTPA MRI proved to be the most useful marker for predicting the therapeutic outcomes of atezolizumab plus bevacizumab therapy in this patient population.

## Author contributions

**Conceptualization:** Heejin Kwon, Yanghyun Baeck, Ilcheol Bark, Jinhan Cho.

**Data curation:** Heejin Kwon.

**Formal analysis:** Heejin Kwon, Eunju Kang.

**Funding acquisition:** Heejin Kwon.

**Investigation:** Heejin Kwon, Sanghyun Kim.

**Software:** Yanghyun Baeck, Ilcheol Bark.

**Supervision:** Heejin Kwon, Sanghyun Kim, Jinhan Cho.

**Visualization:** Yanghyun Baeck.

**Validation:** Sanghyun Kim.

**Writing – original draft:** Heejin Kwon, Ilcheol Bark.

**Writing – review & editing:** Jinhan Cho.
